# Draft Genome Sequences of Two *Vibrio parahaemolyticus* Strains Associated with Gastroenteritis after Raw Seafood Ingestion in Colorado

**DOI:** 10.1128/genomeA.01387-17

**Published:** 2018-01-18

**Authors:** Sonya A. Trinh, Semen A. Leyn, Ivan D. Rodionov, Adam Godzik, Karla J. F. Satchell

**Affiliations:** a Division of Infectious Diseases, Department of Medicine, Northwestern University Feinberg School of Medicine, Chicago, Illinois, USA; b Sanford Burnham Prebys Medical Discovery Institute, La Jolla, California, USA; c Canyon Crest Academy, San Diego, California, USA; d Department of Microbiology-Immunology, Northwestern University Feinberg School of Medicine, Chicago, Illinois, USA

## Abstract

*Vibrio parahaemolyticus* is a Gram-negative pathogen associated with gastrointestinal and wound infections after exposure to raw seafood or contaminated waters. We report here the whole-genome sequences of two stool isolates (CDC-AM50933 and CDC-AM43539) from patients in Colorado presenting with gastroenteritis after ingesting raw seafood.

## GENOME ANNOUNCEMENT

*Vibrio parahaemolyticus* is a halophilic Gram-negative organism found in marine and estuarine environments worldwide (1). The pathogen causes the following three types of infections in patients with exposure to raw or undercooked seafood or contaminated seawaters: gastroenteritis, wound infections, and septicemia (2). Major *V. parahaemolyticus* virulence factors include thermostable direct hemolysin (TDH), type III secretion systems (TTSS1 and TTSS2), and type VI secretion systems (T6SS1 and T6SS2) (3–5). According to the Cholera and Other Vibrio Illness Surveillance (COVIS) system established by the CDC, the incidence of *V. parahaemolyticus* per 100,000 population increased from 0.01 to 0.13 in the United States from 1996 to 2010 (6). The increasing incidence of *V. parahaemolyticus* may be related to climate change, as warming seawaters provide the optimal growth environment for the pathogen (7).

In this paper, we describe the complete genome sequence of two clinical *V. parahaemolyticus* strains isolated from stool cultures of patients presenting with gastroenteritis after eating raw seafood in Colorado, CDC-AM50933 and CDC-AM43539. Both isolates were originally reported to the COVIS as *Vibrio vulnificus*; however, the strains were identified as *V. parahaemolyticus* by 16S rRNA sequencing. The isolates were further confirmed as *V. parahaemolyticus* on CHROMagar Vibrio (CHROMagar Microbiology, France) and TCBS (thiosulfate-citrate-bile-sucrose) agar (BD, USA).

Strain CDC-AM50933 was isolated from a stool culture of a 59-year-old healthy male who presented with *V. parahaemolyticus* gastroenteritis in Colorado. He had a history of consuming raw clams, mussels, oysters, and shrimp and cooked crab, lobster, and crawfish. He presented as an outpatient with fevers, nausea, vomiting, abdominal pain, and more than 10 episodes of diarrhea. The antibiotic treatment received was not reported to the COVIS. The symptoms lasted for 6 days with no other adverse reactions. Strain CDC-AM43539 was isolated from a stool culture of a 69-year-old male with a history of gastric bypass surgery who presented with *V. parahaemolyticus* gastroenteritis in Colorado. He had a recent history of ingesting crab, shrimp, and raw oysters. The patient presented with abdominal cramps and more than 10 episodes of diarrhea. During his 26-day hospitalization, he was treated with a course of doxycycline and metronidazole.

Genomic DNA was extracted using the Maxwell 16-cell DNA purification kit, and DNA libraries were prepared using the Illumina Nextera XT DNA library preparation kit according to the manufacturer’s protocol. An average coverage of 91× was achieved using HiSeq 4000 paired-end 150-bp sequencing at the Institute for Genome Sciences at the University of Maryland School of Medicine. Assembly was performed using SPAdes version 3.10.0 (8), and annotation was added by the NCBI Prokaryotic Genome Annotation Pipeline as implemented at the PATRIC Bioinformatics Database and Analysis Resource Center (9). Both strains CDC-AM50933 and CDC-AM43539 had genes encoding virulence factors TDH, TTSS1, and TT6SS2.

### Accession number(s).

The whole-genome sequences reported here have been deposited at DDBJ/ENA/GenBank under the accession numbers NPOM00000000 (CDC-AM50933) and NPOL00000000 (CDC-AM43539).
